# Supplementary material for: cAMP Compartmentalisation in Human Myometrial Cells
Source: Cells. 2023 Feb 24;12(5):718. doi: 10.3390/cells12050718 (PMC10001376; doi:10.3390/cells12050718)
Supplement: Supplementary file 1 [file cells-12-00718-s001.zip › cells-2227411-supplementary.pdf]

Supplementary information and data

Term no labour samples, n	
Sample number, n	20
Maternal age, years	35.4 ± 1.0
Maternal BMI, kg/m^2	24.4 ± 1.0
Gestational age at delivery, weeks	38.5 ± 0.2
Parity, number	1 ± 0.1

Table S1: Demographics for term no labour samples. Data expressed as mean ± standard error of the mean (SEM).

Antibody	Predicted molecular weight of targeted protein (kDa)	Source and Catalog number
AKAP79	79	New England BioLabs Ltd., D28G3, Herts, UK
B2 Adrenergic receptor	46	Abcam Ltd., ab61778, Cambridge, UK
EP2 receptor	40	Abcam Ltd., ab167171, Cambridge, UK
GAPDH	38	Millipore, MAB374, Watford, UK
PDE4B	100	University of Glasgow, a gift, not commercially available
Connexin-43	39-43	Cell Signalling Technology, 3512
Anti-goat IgG, HRP-conjugated	n/a	Dako/Agilent, P0449
Anti-mouse IgG, HRP-conjugated	n/a	Cell Signalling Technology, 7076
Anti-rabbit IgG, HRP-conjugated	n/a	Cell Signalling Technology, 7074

Table S2: Primary and secondary antibodies. Includes source and catalog number.

Name	Primer sequence (5′–3′)	GenBank/EMBL accession no.
AKAP79	F:cggaaagatggtgatgaggt<br>R:tactggctgctgatggtctg	NM_004857
GAPDH	F:tgatgacatcagaaggtggtgaag<br>R:tccttggaggccatgtaggccat	BC014085
OTR	F:agaagcactcgcgcctctt<br>R:aggtgatgtcccacagcaact	NM000916
PDE4B	F:ccctgttgtccagtccaact<br>R:tgcagactctcacggtgaac	NM_001037339
Cx43	F:tggattcagcttgagtgctg<br>R:ggtcgctctttcccttaacc	BC026329

Table S3: Primer sequences. Includes GenBank/EMBL accession number.

Epac-S^{H187} (ISO 1nM) Figure 2A, 3B & 9A	Epac-S^{H187} (ISO 1μM) Figure 2A, 3B & 9A	Epac-S^{H187} (PGE2 1μM) Figure 2B, 3B & 9C	Epac-S^{H187} (PGE2 30nM) Figure 2B, 3B & 9C	AKAP79-CUTie (ISO 1nM) Figure 2C, 3B & 9B	AKAP79-CUTie (ISO 1μM) Figure 2C, 3B & 9B	AKAP79-CUTie (PGE2 1μM) Figure 2D, 3B & 9D	AKAP79-CUTie (PGE2 30nM) Figure 2D, 3B & 9D
D30 = 3	D36 = 3	D30 = 2	D41 = 5	D31 = 3	D36 = 3	D30 = 2	D41 = 2
D31 = 4	D39 = 3	D31 = 3	D42 = 3	D33 = 3	D37 = 4	D31 = 2	D42 = 3
D33 = 4	D40 = 4	D33 = 4	D43 = 3	D34 = 4	D40 = 4	D33 = 4	D43 = 2
	D41 = 4	D34 = 4	D47 = 8		D41 = 3	D34 = 5	D47 = 2
	D42 = 3		D48 = 6		D42 = 3		D48 = 3
	D43 = 3		D49 = 5		D43 = 2		D49 = 3
	D47 = 7		D50 = 12		D47 = 2		D50 = 6
	D48 = 5		D51 = 3		D48 = 4		D51 = 3
	D49 = 5		D55 = 7		D49 = 7		D55 = 2
	D50 = 18		D56 = 5		D50 = 6		D56 = 8
	D51 = 4		D57 = 9		D51 = 2		D57 = 5
	D55 = 8		D58 = 10		D56 = 2		D58 = 4
	D56 = 3				D57 = 4		
	D57 = 9				D58 = 10		
	D58 = 9						
total = 3 donors	total = 15 donors	total = 4 donors	total = 12 donors	total = 3 donors	total = 14 donors	total = 4 donors	total = 12 donors

Table S4: Cell numbers per individual donor for the cAMP responses to ISO or PGE2 in the cytosol and at the plasmalemma of HPMCs.

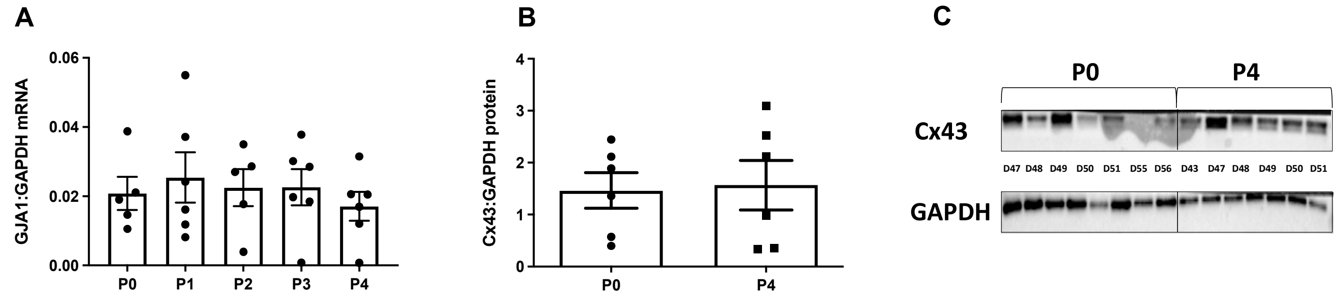

Figure S1: Connexin-43 mRNA & protein expression in term no labour HPMCs from p0 to p4. Isolated HPMCs were cultured to 80% confluence at p0 and passaged to p4. At each passage, RNA was extracted and synthesised to cDNA for subsequent qPCR for GJA1 (**A**) and protein was extracted and quantified using western blotting. Densitometric analysis for connexin-43 (**B**) with representative blot (**C**). Data normalised to GAPDH and expressed as mean; error bars represent SEM; Normality was tested using Kolmogorov-Smirnov test. For mRNA, data were analysed using a one-way ANOVA followed by a Turkey's multiple comparisons test. For protein, data were analysed using a paired t-test. Each data point or protein band indicates HPMCs from individual donors.mRNA [n=5-6], protein p0 [n=7]; protein p4 [n=6].

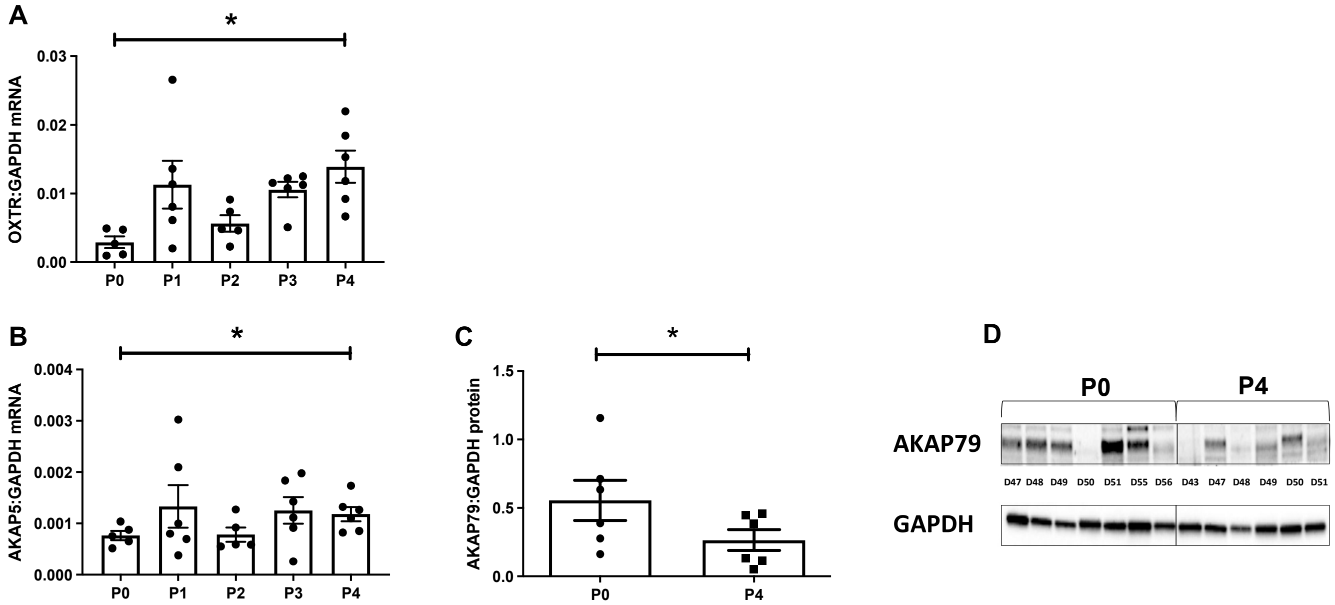

Figure S2: OXTR and AKAP5 mRNA expression and AKAP79 protein levels in term no labour HPMCs from p0 to p4. Isolated HPMCs were cultured to 80% confluence at p0 and passaged to p4. At each passage, RNA was extracted and synthesised to cDNA for subsequent qPCR for OXTR (**A**) and AKAP5 (**B**). Protein was also extracted and quantified using western blotting at each passage. Densitometric analysis for AKAP79 (**C**) with representative blot (**D**). Data normalised to GAPDH and expressed as mean; error bars represent SEM; Normality was tested using Kolmogorov-Smirnov test. For mRNA, data were analysed using a one-way ANOVA followed by a Turkey's multiple comparisons test. For protein, data were analysed using a paired t-test. Each data point or protein band indicates HPMCs from individual donors.mRNA [n=5-6], protein p0 [n=7]; protein p4 [n=6-7]. * = 0.05 < p < 0.01.
